# The Adirondacks in Winter for Tubercular Patients1Read at the thirtieth annual meeting of the Medical Association of Central New York at Buffalo, October 19, 1897.

**Published:** 1898-04

**Authors:** Sargent F. Snow

**Affiliations:** Syracuse, N. Y.


### THE ADIRONDACKS IN WINTER FOR TUBERCULAR PATIENTS.[1]

By SARGENT F. SNOW, M. D., Syracuse, N. Y.

THE favorable discussion which followed a paper before this society in Syracuse two years ago, on Aural, nasal and laryngeal tuberculosis, with special reference to the Adirondacks as a winter health resort,—the results of later observation and the fact that, to date, no treatment devised for these affections of the respiratory tract acts so well as the climatic treatment,—encourages me to present this, as a supplement to the first, under the title The Adirondacks in winter for tubercular patients.

As a review of the above-mentioned article, I will quote from the conclusions therein: (See Buffalo Medical Journal, December, 1895.)

1. That we have here at home, in the Adirondacks of our own state, localities where many tubercular patients may find the climatic and physical environment required.

2. That the patients do equally as well in fall and winter and gain more than in the summer months.

These conclusions were arrived at after a careful scrutiny of results to that time. To sum up later experiences and avoid too much detail, an abstract of a few cases will perhaps be most expedient.

Prominently, I would mention a young woman who came under my notice in October, 1895. Examination showed marked tubercular ulcerations along the inner side of the left arytenoid cartilage, together with some dulness and respiratory murmur in upper lobe of right lung. The general appearance of this patient was such as we often meet, being pale and careworn, with history of almost constant poor health and throat trouble for years. She

---

1. Read at the thirtieth annual meeting of the Medical Association of Central New York at Buffalo, October 19, 1897.

began her residence in the Adirondacks December, 1895, staying there until May 20th of the next year, when she returned home a most perfect picture of health, weighing 140 pounds, muscles hard, good color, no rise of temperature, laryngeal ulcerations removed, with only a slight thickening of the membranes in that region as a reminder of her former trouble.

This young woman, while in the woods, began a vigorous routine of life as soon as her temperature became normal, taking long tramps on snow shoes, and improving every opportunity to be out of doors. The gain of weight was about twenty pounds, which, together with the superb physical appearance, has remained permanent until the present writing. She resumed her position as bookkeeper, which she has filled without interruption.

This case, I believe, should be looked upon as exceptional and should not be taken as a safe instance for guidance, for, as a rule, it does not seem best for such patients to so soon resume their ordinary routine of life.

My practice being limited to ear, nose and throat affections, makes it true that most of the tubercular cases observed are in the first stage, but there have been exceptions, one of which I will mention. A well-known homeopathic physician of Syracuse called to see me July 2, 1896, complaining of irritable pharynx with distressing cough. Upon inquiry I found that one year previous he had had considerable pulmonary hemorrhage, but had been able to continue his work. For four months he had been losing weight, throat had been sore and much mucus had been expectorated. I soon became convinced that his local catarrhal manifestations were of little importance compared to the constitutional trouble. An examination of sputum was made, with report that it was loaded with tubercular bacilli and fibrous tissue. Physical examination of the lungs revealed an area of marked dulness with soft râles, which area before he left town was well broken up and a cavity forming.

I kept close watch of this patient, spending about two weeks as a near neighbor of his in the woods the following August, and was very much impressed with the gravity of the situation. The beneficial effects of his surroundings appeared to be almost overcome by the activity of the tubercular process. During the first three weeks, instead of gaining, he seemed to have lost ground, and when I met him was barely able to drag himself about. A more careful regulation of his mode of life, with enforced rest, was followed by some improvement, though no decided change for the better was shown, all symptoms indicating a gradual and continuous destruction of lung tissue, but with the advent of cold weather he began to improve and was soon able to do a little practice in the community where he was staying. This improvement continued in a marked manner until the warmer months, when there was a tendency to drop back some, but his close and careful attention to diet, habits, etc., has kept him during the summer months of this year in a very good condition,—being able to carry on quite a large practice. I am assured by letter and other reliable sources that he feels and seems almost well, and very content to spend another winter in the same region,—a marked testimonial to the value of the climate in the colder months.

As a parallel to the above, a brief mention of two other cases might be made ; one, a young man, who boarded at the same place with the doctor and who, when I first met him, appeared to be in about the same condition, but who before two weeks' stay in the woods was up, had clearly made more gain in strength and whose cough was much more diminished. This individual was like the doctor, in the second stage of pulmonary tuberculosis, but, I am informed, had continued to improve until his return home to Buffalo in October. The details of the succeeding development of the case I have been unable to learn, except that he spent the winter in Colorado, returned home in the spring and soon died.

The second parallel case, parallel because he came under my care and went to the woods about the same time as my medical friend, but who appeared as a much more favorable case, spent six weeks in the Adirondacks, returned home showing marked improvement, having gained twelve pounds in weight, normal temperature and an outward appearance of complete restoration of health.

Being convinced that it would not do to resume his old position in business, he started for Denver, where he stayed until early summer. To my disappointment and though he had been advised to go there by several eminent men, the climate did not agree with him and he came home again to this state much worse and well along in the second stage. It surely seems that either of these two cases would have fared better had they let well enough alone and spent the fall, winter and spring with the doctor in the Adirondacks.

Experience proves it to be dangerous for such patients to migrate to a dryer or warmer climate, even during the breaking up of spring time, as advised by some, for the chances are that in their journey they will stop off for a visit at their old home and thereby renew the tubercular activity sufficient to more than overcome the benefit they would get from the change. A continuous residence until the disease is well under subjection is almost imperative.

The foregoing facts are submitted as the gist of an impartial observation of the great benefit given these patients by the Adirondack air in winter as well as in summer, and if the experience of the medical men of central and western New York be, or should become, along the same line, we can look into the future and see great changes. The state authorities will learn that those magnificent virgin forests have a value beyond that of fostering game and protecting the head waters of rivers—that of helping to make complete a divine gift to humanity—an all-the-year-round sanitarium for consumptives.

We can perhaps look again and further into the future and see the hospitals of adjacent cities relieved of their depressing and germ-producing tubercular cases, because of auxiliary hospitals or sanitariums, under charitable or municipal control, located at some convenient spot within the woods. If we look again, we may see these same auxiliaries endowed and enlarged by some of our philanthropic capitalists, the dreaded disease robbed of virulence and cheated of many victims. Such philanthropy is well illustrated in the recent development of the Loomis Sanitarium, at Liberty, Sullivan county, N. Y.

Pardon this entrance into the realm of prophesy. The present commands our attention. If the medical profession looks upon the propositions set forth in this paper as true, the future will take care of itself.

Eminent authorities have long recognised the value of the mountainous regions of New York state as a summer resort for tubercular patients, and those who have tried them as a winter sanitarium will, we believe, fully agree, when I assert that they are 25 per cent. more valuable in the colder months.

No fear need be entertained because of the rigorous climate, for the invigorating effects appear to compensate. If we expect permanent results, only those in the first or early part of second stage should be sent and even the best of these may not be able to again live in a moisture-laden atmosphere.

204 East Jefferson Street.

### DISCUSSION.

Dr. MOONEY, of Syracuse : I would like to add the history of one case similar to these reported. One afternoon a married woman, thirty years old, came from northern Michigan to her former home, Syracuse, suffering with incipient phthisis and the sputum showing plenty of bacilli. She was sent to the Adirondacks at my suggestion and remained there until the 1st of May. She weighed ninety-four pounds when she went there and on her return the 1st of May weighed 116, the most she had ever weighed in her life, and microscopic examination of the sputum showed the absence of bacilli. She has remained home ever since pursuing her usual occupation of housework, apparently in perfect health.

Dr. ROCHESTER, of Buffalo : I have been in the habit of sending cases to the Adirondacks for the last ten years and I must say that I shall continue to send them there in increasing numbers ; and not merely those in the first stage, or even the beginning of the second stage, although, of course, those cases are the ones that get well ; I think that even advanced cases of tuberculosis are benefited by a stay in the Adirondacks for as long a period as they can afford to be there. Those advanced cases are, of course, not sent there with the chance of recovering. I have three cases in mind at present that were sent some time ago who have recovered entirely, one of whom has gone west to live and one of whom has returned to Buffalo to live and remains without evidences of the disease. I have this year sent five cases to the Adirondacks and all of them are improving very materially. The two particularly that were in the first stage, early in the first stage, were in the incipiency, have right straight through the summer and this fall constantly gained in weight and improved in every respect ; the fever has left them entirely ; the physical signs have almost disappeared. One case I returned here for a week a short time ago and one case has returned not benefited, who was an advanced case. There is one thing, however, about the Adirondacks that I think is important for us to bear in mind and a warning that we must give our patients when we send them there—that is, the importance of staying there.

One of the advantages of the Adirondacks is that it is near and our patients can go there without very great expense. It is also one of its greatest disadvantages that it is near, because they can come home and they think they can run down for a little excursion, a little stay of a week or ten days, or something of that sort. That is where the bad results come in. If they could go there and stay and we could insist upon their staying until they were discharged by the physician in charge in the Adirondacks, the final results would be much better than if they went there and came back when they felt well, for so many of them come back feeling well before they are well, that in the course of six months or so they are worse off than when they first went. One other thing further I would like to speak about is the manner of making our diagnoses of tuberculosis before the tubercle bacilli appear in the sputum. I think we ought to do that and that is a point upon which we as scientific observers of the present day are getting a little bit below our fathers in acuteness of observation. Tuberculosis ought to be diagnosticated before the process in the lung has proceeded so far that the bacilli appear in the sputum, and it strikes me that careful study of cases in all their aspects, of not merely physical signs, but the symptoms leading up to, and taking into consideration very careful exploration of the chest, should enable us to make our diagnosis of tuberculosis before the tubercle bacilli appear in the sputum.

Dr. Ely, of Rochester : I listened with great interest to this paper and the remarks of Dr. Rochester and others. I can only add this to what has been said : that I have been able to make diagnoses in some cases by the microscope before the physical signs were clearly apparent and I think that consumption is more curable today than it ever has been before, because of the capacity for its early appreciation and the recognition that climatic change is the essential thing. I am sure that cases can be diagnosticated before tubercle bacilli appear in the sputum, but I question, sir, whether there are not some cases which can be diagnosticated by the microscope when the physical signs upon the examination of the chest would leave the physician in doubt.

Dr. Elsner, of Syracuse : The paper is full of interest and it strikes me that the discussion which it is bringing out is equally interesting and equally important. One of the greatest problems which we have to solve today is the early recognition of pulmonary tuberculosis and I am very happy to hear these two gentlemen who have just spoken speak of the recognition of this disease early, because therein lies the safety of all of these patients. It is important in these cases to recognise the disease before the breaking down of tuberculous infiltrates which set loose these tubercle bacilli. It is important for us to recognise miliary tuberculosis in its incipiency and the most grave forms of miliary tuberculosis, you all know, do not for a long time give the tubercle bacillus in the sputum. For that reason I think that everything which tends towards the diagnosis of incipient phthisis is of the greatest importance and if we occasionally make a mistake and diagnose a condition as being tuberculous which is not tuberculous, the mistake does no harm. On the other hand, if we fail to recognise these diseases in their incipiency we do great mischief. The time when the tubercle bacillus shows itself in the sputum varies materially with the different forms of pulmonary tuberculosis. You will frequently have cases of pulmonary tuberculosis which you will examine until the end almost and the sputum will fail to show the presence of the bacilli. Those are the cases where you have the nodulary form of tuberculosis, not the infiltrating form and the form which does not break down. This appears to me to be one of the most important points which has been brought out in this discussion and I wish to emphasise very emphatically what both Dr. Rochester and Dr. Ely state in connection with it.

Dr. LOVELAND, of Clifton Springs: The subject of the Adirondacks in winter as a resort for consumptives has come within my observation on several occasions and I quite agree with what Dr. Snow has said, that it is, in my experience among my patients, more beneficial in the winter than in the summer. One point I felt like speaking of in connection with the subject, which seems to me in many cases to be overlooked, is the necessity of getting the patient started upon a diet which will be sufficiently nutritious and getting them, so to speak, in the habit of eating enough nutritious food before they go at all. I have seen patients sent for climatic change and given creosote in large quantities and all the various things that are all right, but nothing said about, for instance, how many eggs or how much milk or how much meat they shall take, and the patient going on with no attention very definitely given to diet, does not do well. The diet is such a strong help in any climatic condition that it should not be overlooked.

Dr. SNOW: There is not much to add, except to thank the gentlemen who have discussed the paper, and place emphasis on the point that the Adirondack climate is of particular value in tubercular cases during the colder months.

